# Computational fluid dynamics evaluation of physician-modified stent graft repair for distal residual stanford type B aortic dissection

**DOI:** 10.1186/s12872-026-06172-2

**Published:** 2026-07-03

**Authors:** Wei Liu, Chi Cui

**Affiliations:** Center of Vascular and Interventional Surgery, Department of General Surgery, The Third People’s Hospital of Chengdu, Affiliated Hospital of Southwest Jiaotong University & The Second Affiliated Hospital of Chengdu, Chongqing Medical University, Chengdu, 610031 China

**Keywords:** Type B aortic dissection, Hemodynamics, Physician-modified stent grafts, Distal tears and residual dissection

## Abstract

**Background:**

Few studies have focused on physician-modified stent grafts (PMEGs) for treating distal residual dissection in type B aortic dissection (TBAD). This study aimed to assess the usage of PMEGs in the treatment of distal residual dissection of TBAD by analyzing the relevant hemodynamical indicators.

**Methods:**

Patients with TBAD underwent thoracic endovascular aortic repair surgery in the first stage, and in the second stage PMEGs were used to repair the residual dissection. Computational fluid dynamics and three-dimensional structural analyses were performed, based on computed tomography angiography datasets. The prognostic post-implantation improvement was studied using both quantitative and qualitative functional analysis.

**Results:**

A total of 30 patients with TBAD were enrolled. Following the PMEGs procedure, peak systolic pressure in all aortic segments trended downward without statistical significance. Overall time-averaged wall shear stress (TAWSS) increased significantly (*P* = 0.007), especially in the S2 segment (*P* = 0.005), while overall oscillatory shear index decreased significantly (*P* = 0.010). Superior mesenteric artery blood flow was significantly higher postoperatively (*P* = 0.005), with no significant differences in other visceral branches. At 1‑year follow-up, false lumen volume was markedly reduced (*P* < 0.001) and true lumen volume increased, suggesting favorable aortic remodeling.

**Conclusions:**

The PMEGs technique effectively ameliorates hemodynamic parameters in patients with residual distal dissection following TBAD. However, long-term follow-up of the increase in TAWSS is still required.

## Introduction

Thoracic endovascular aortic repair (TEVAR) is the preferred treatment for type B aortic dissection (TBAD) [1, 2]. However, standard TEVAR mainly addresses proximal tears, leaving distal tears and residual dissection to heal without direct surgical intervention [3, 4]. Growing evidence suggests that these untreated conditions may benefit from surgical management. Distal aortic dilatation has been reported in approximately 25–40% of cases, with a reported 3-year mortality of up to 36% [5–7]. Therefore, residual aortic dissection resulting from TEVAR, including both the abdominal aorta and iliac artery segments, remains an unresolved clinical issue.

Open surgery is the gold standard for treating distal TBAD, yet it is an invasive procedure with high mortality and complication rates [8, 9]. At present, there is no consensus on the optimal endovascular approach for managing residual aortic dissection due to TBAD. However, complete isolation and repair of distal tears and residual dissection using fenestrated and branched stent grafts is widely regarded as the most effective strategy [4, 6, 10].

There is currently no standardized treatment model for distal residual dissection of TBAD stent-graft repair. Whilst physician-modified stent grafts (PMEGs) have emerged as a promising option [11], their application in distal TBAD dissection is limited in the literature, and their efficacy has yet to be fully determined.

This study presents a retrospective analysis of patients with distal residual dissection of TBAD who were treated with PMEGs at our institution. Computational fluid dynamics (CFD) was used to assess hemodynamic changes before and after the procedure. The resulting detailed morphological and functional insights provide a valuable reference for evaluating PMEGs in terms of their therapeutic efficacy, thereby potentially offering guidance for future clinical management of distal residual dissection of TBAD.

## Methods

### Patients

We reviewed patients with TBAD who were admitted to our center between January 2019 and February 2025. Eligible patients had undergone TEVAR for proximal entry tear closure during their initial hospitalization and later received PMEGs for distal residual dissection during a second hospitalization. Data were collected in March 2026 and were fully anonymized prior to receiving them. The criteria for patients to undergo PMEGs included complications of residual aortic dissection during follow-up, such as organ ischemia, progression of the false lumen (FL) (more than 5 mm within 6 months), and posterior aortic aneurysm formation (greater than 5 cm). The technical details of PMEGs in this study have been described in detail in our previous publication [3]. Patients with incomplete data were excluded from the study. A total of 30 patients met the inclusion criteria. The study was approved by the Ethics Review Committee of Chengdu Third People’s Hospital; because of its retrospective design, the requirement for patient informed consent was waived by the same committee.

### Image acquisition and geometry reconstruction

**Fig. 1:**
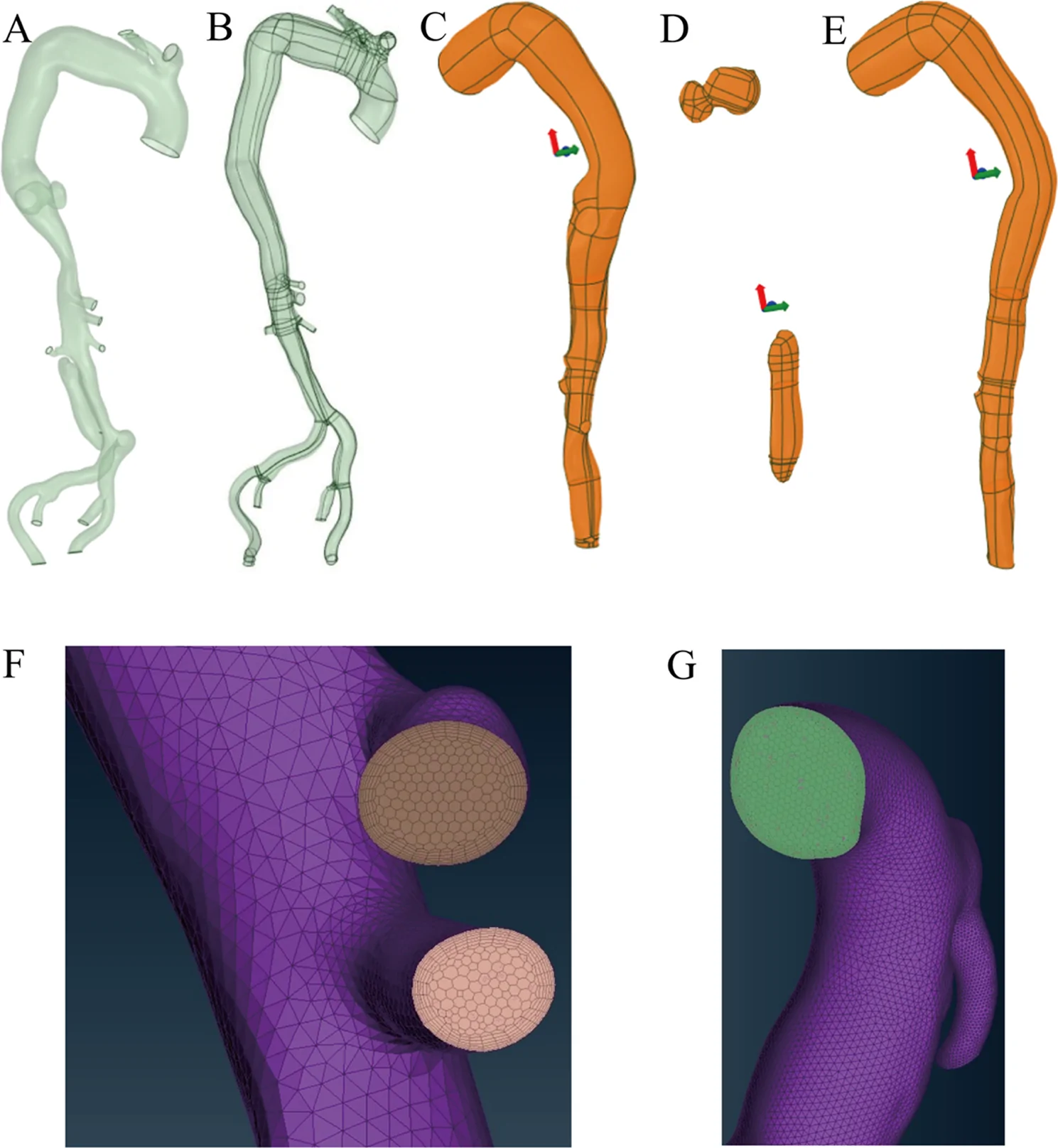
3D reconstruction of the oarta model before (**A**) and after (**B**) surgery; segmentation of true (**C** and **E**) and false (**D**) lumens by SCDM and calculation of volumes; ANSA grid processing (**F** and **G**)

Each patient underwent two aortic computed tomography angiography (CTA) scans, the first occurring before surgery and the other at the 1-year follow-up point. All CTA datasets were acquired using a dual-source CT scanner (Brilliance iCT256, Royal Philips, The Netherlands). Pre- and postoperative DICOM data were exported for analysis. Three-dimensional (3D) aortic models were reconstructed from the CTA data using the medical imaging software DetecModeling (Boea Wisdom Technology, Hangzhou, China). The reconstructed 3D aortic models before and after surgery are shown in Fig. 1A and B, respectively.

### True lumen and false lumen volume measurements

True lumen (TL) and FL volumes were estimated using the reconstructed 3D models. Post-processing was performed with CFD-Post (version 19.0, ANSYS, USA), where the vascular model was segmented and sectioned to compare and measure the volumes. Subsequently, volume measurements were obtained using DetecFluid (Boea Wisdom Technology, Hangzhou, China) (Fig. 1C-E).

### Numerical models

The Navier-Stokes (N-S) equations were numerically solved via the finite-volume CFD solver DetecFluid (Boea Wisdom Technology, Hangzhou, China). Blood was treated as a Newtonian fluid with a density of 1060.00 kg/m³ and a dynamic viscosity of 0.00400 Pa·s. The inlet flow waveform was derived from the literature [12]. The vessel wall was set to a rigid boundary with the no-slip condition applied. Each outlet was coupled with the three-element Windkessel model (WK3) [13–15].The volume and surface mesh size ranged from 0.5 to 2 mm, with a branch pipe size of 1 mm and a main pipe size of 2 mm, while the branch junction feature size was set to 0.5 mm. A boundary layer consisting of 7 layers was applied, with a growth rate of 1.2 and an initial layer thickness of 0.12 mm (Fig. 1F-G). The flow was modeled as incompressible laminar flow with velocity inlet boundary conditions. Simulations were performed over two cardiac cycles, each with a period of 0.8 s, and data from the second, more stable cycle were used for analysis.

### Analysis of hemodynamic parameters

**Fig. 2:**
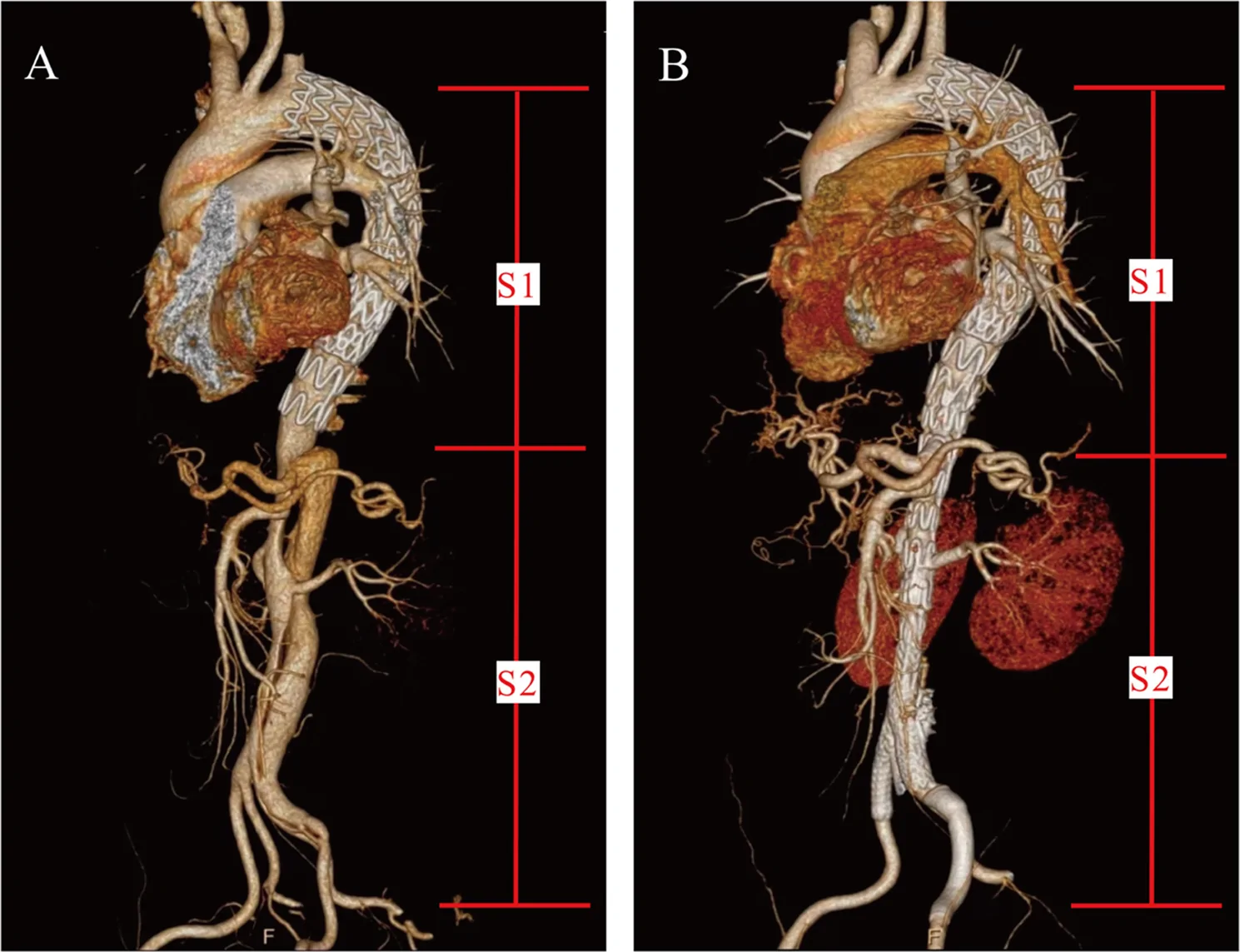
Computed tomography angiogram (CTA) of the patient before (**A**) and after (**B**) physician-modified stent grafts (PMEGs). S1, the stent-covered segment after thoracic endovascular aortic repair (TEVAR) surgery; S2, the distal residual dissection segment before the PMEGs procedure and the stent-covered segment after the PMEGs procedure

Both qualitative and quantitative analysis methods were used to evaluate hemodynamic changes before and after PMEG implantation. Qualitative evaluation included aortic peak systolic pressure and wall shear stress (WSS)-related parameters, including time-averaged wall shear stress (TAWSS), oscillatory shear index (OSI), and relative residence time (RRT). For quantitative analysis, the aorta was divided into two segments according to the first operation (S1 segment) and the second operation (S2 segment) (Fig. 2A-B). Within these segments, aortic pressure, average WSS-related parameters, and blood flow in the four abdominal branches (the celiac trunk, superior mesenteric artery, and bilateral renal arteries) were calculated and compared before and after PMEG treatment.

### Statistical methods

Continuous variables with a normal distribution were expressed as mean ± standard deviation, while non-normally distributed variables were represented by median and interquartile range. Categorical variables were presented as frequencies and percentages. A two-sided P value less than 0.05 was considered statistically significant. All analyses were performed using SPSS version 26.0 (IBM Inc., Armonk, NY).

## Results

### Baseline characteristics and clinical outcomes

As shown in Tables 1 and 30 patients were included in this study according to the inclusion criteria, including 28 males. The median age was 64.5 years with a range of 44.5–71.8 years. All patients had a history of hypertension. Among them, 14 patients (47.6%) suffered from chronic obstructive pulmonary disease (COPD), 6 patients (20%) had a history of stroke, and 6 patients (20%) had diabetes. Four patients (13.3%) had a history of renal insufficiency, while hyperlipidemia and myocardial infarction were observed in 1 patient (3.3%). Patients identified as suitable for PMEG intervention presented with FL progression (83.3%) and post-dissection aneurysm (16.7%). Technical success was achieved in all 30 patients (100%). There were no 30-day deaths, stroke, spinal cord ischemia, myocardial infarction, or organ failure. Type II endoleak occurred in 7 patients (23.3%), with no Type I or Type III endoleak. Reintervention was required in 4 patients (13.3%), all of whom underwent reintervention for persistent Type II endoleak. No target vessel occlusion or 1-year aortic-related mortality was observed during follow-up.

**Table 1:** Initial patient characteristics and clinical outcomes

Variables	Value
No. of patients	30
Age, years	64.5 (44.5–71.8)
Male/female	28/2
Comorbidities	
Hypertension (n, %)	30, 100
Diabetes (n, %)	6, 20
Stroke (n, %)	6, 20
Renal insufficiency (n, %)	4, 13.3
Hyperlipidemia (n, %)	2, 6.7
Miocardial infarction (n, %)	1, 3.3
COPD (n, %)	14, 46.7
Reasons of PMEGs	
False lumen progress (n, %)	25, 83.3
Post-dissection aneurysm > 5 cm (n, %)	5, 16.7
Clinical outcomes	
Technical success (n, %)	30, 100
30-Day mortality (n, %)	0, 0
Stroke (n, %)	0, 0
Spinal cord ischemia	0, 0
Myocardial infarction (n, %)	0, 0
Organ failure (n, %)	0, 0
Endoleak	
Type Ⅰ (n, %)	0, 0
Type Ⅱ (n, %)	7, 23.3
Type Ⅲ (n, %)	0, 0
Reintervention (n, %)	4, 13.3
Target vessel occlusion (n, %)	0, 0
1-year aortic-related mortality (n, %)	0, 0

*COPD* chronic obstructive pulmonary disease, *PMEGs* physician-modified stent grafts

### Pressure fields

**Fig. 3:**
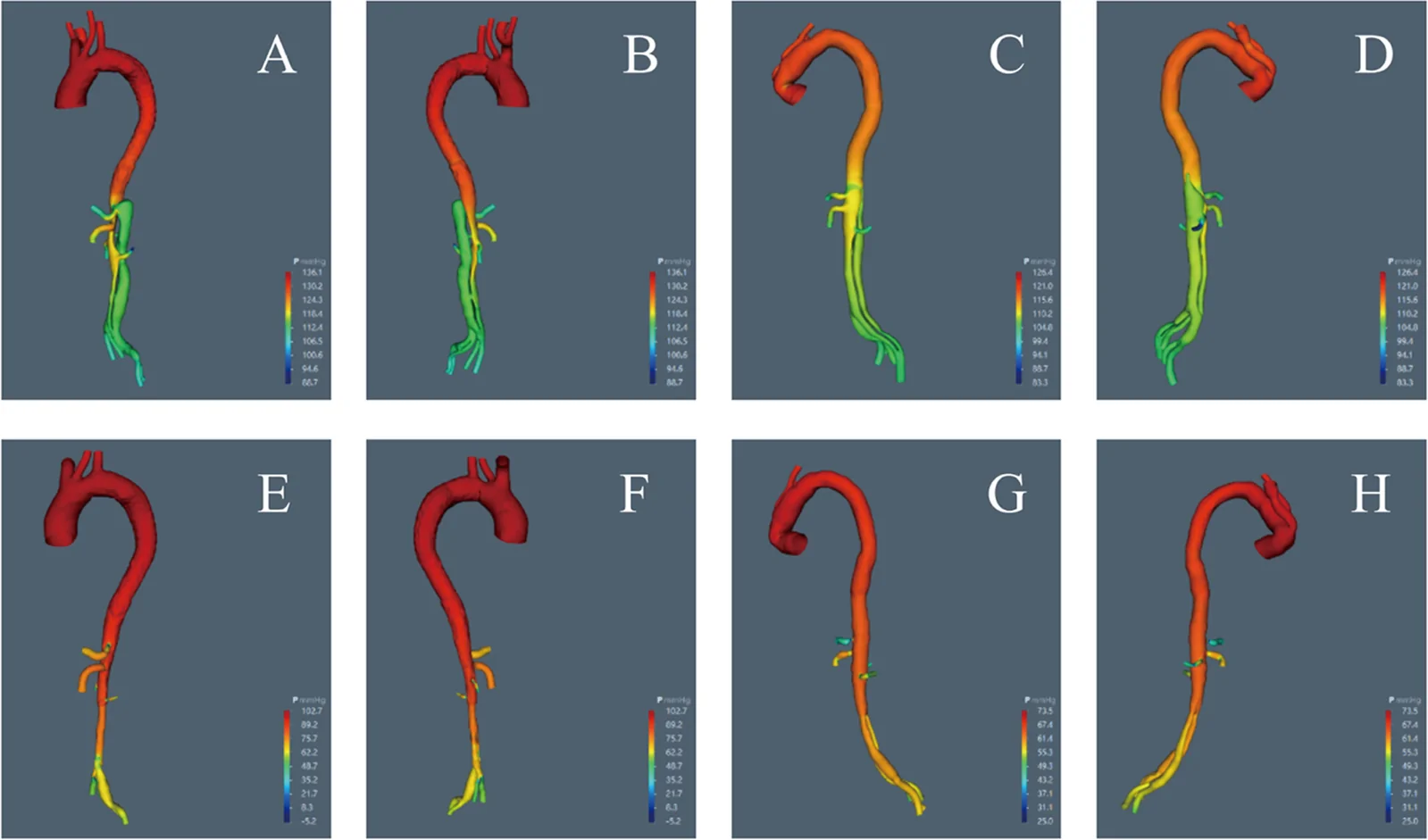
Pressure distributions of two patients preoperative (A-D) and postoperative (E-H) physician-modified stent grafts (PMEGs) surgery

Figure 3 illustrates the pressure distribution at peak systolic blood pressure before and after PMEG surgery. The pressure color scales were standardized across all cases to facilitate visualization and direct comparison. Before the surgery, aortic pressure was higher and unevenly distributed. After PMEG treatment, overall aortic pressure decreased significantly and became more evenly distributed. Meanwhile, pressure within the four branches of the visceral area of the abdominal aorta (including the celiac trunk, superior mesenteric artery, and bilateral renal arteries) showed an increase compared with the preoperative state.

### WSS-based parameter analysis

**Fig. 4:**
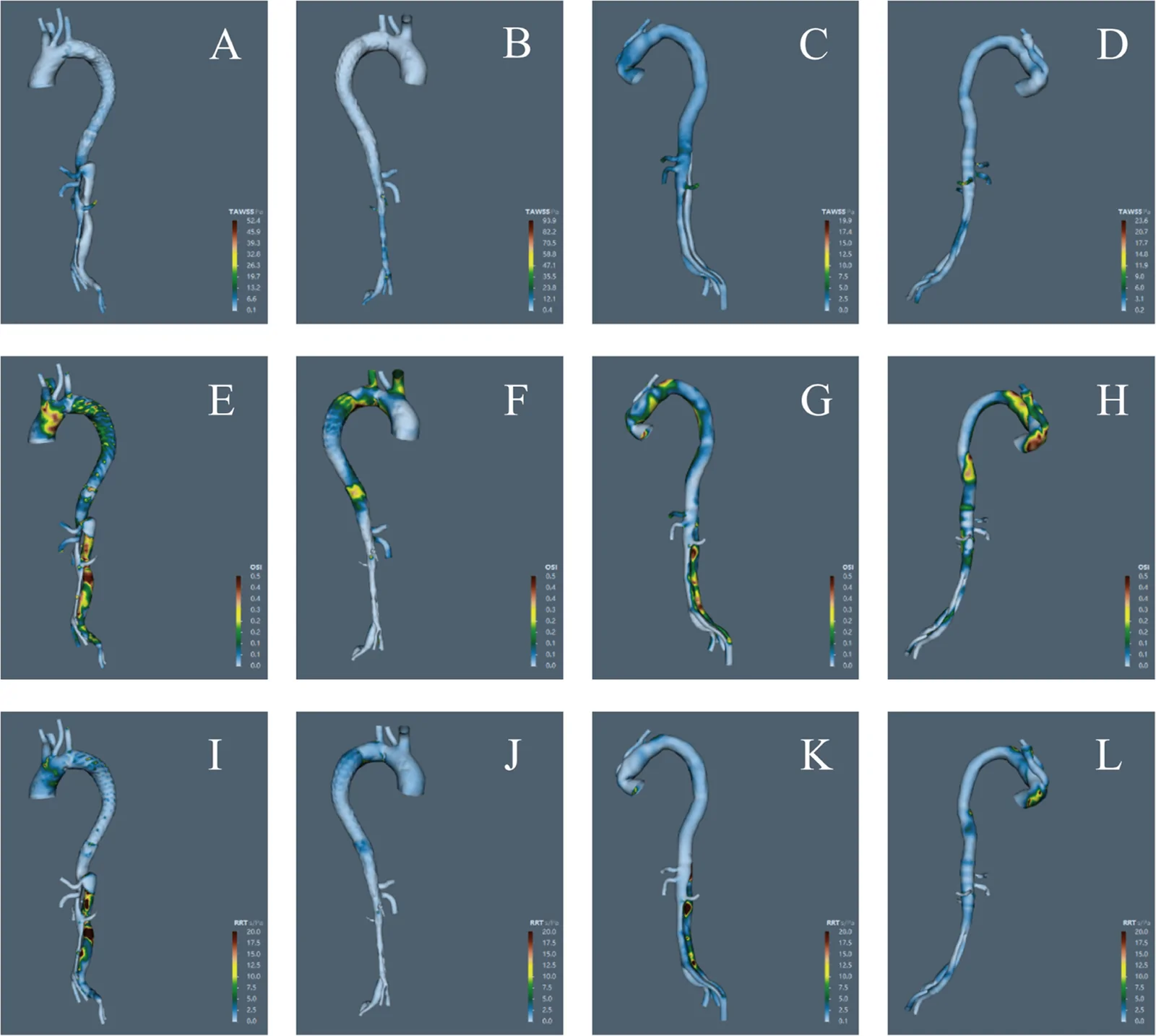
Preoperative and postoperative changes of wall shear stress related parameters. Distribution of time-averaged wall shear stress (TAWSS) before (**A** and **C**) and after (**B** and **D**) surgery. Changes of oscillatory shear index (OSI) before (**E** and **G**) and after (**F** and **H**) surgery. Preoperative (**I** and **K**) and postoperative (**J** and **L**) changes of relative time (RRT)

WSS-related parameters are crucial for the hemodynamic analysis of vascular remodeling following stent implantation. Figure 4 shows the distributions of TAWSS, OSI, and RRT before and after surgery. Before the surgery, increased TAWSS was observed above the celiac artery and at the rupture site of the abdominal aorta (Fig. 4A C). After stent implantation, TAWSS in these areas was significantly reduced (Fig. 4B and D). Figure 4E-H show contour maps of OSI, whereby preoperatively, OSI was significantly higher in the FL, while postoperatively, OSI within the aorta decreased significantly and became more evenly distributed. Figure 4I-L show cloud maps of RRT. Prior to surgery, RRT was relatively low in the TL but significantly higher in the FL of the thoracic and abdominal aortas. After the surgery, RRT became more uniformly distributed within the stented segments of the thoracic and abdominal aorta, although scattered areas of high RRT persisted. No significant changes in WSS-related parameters were observed in the visceral branch region of the abdominal aorta before and after the four-branch surgery.

### Quantitative analysis of hemodynamic status

**Fig. 5:**
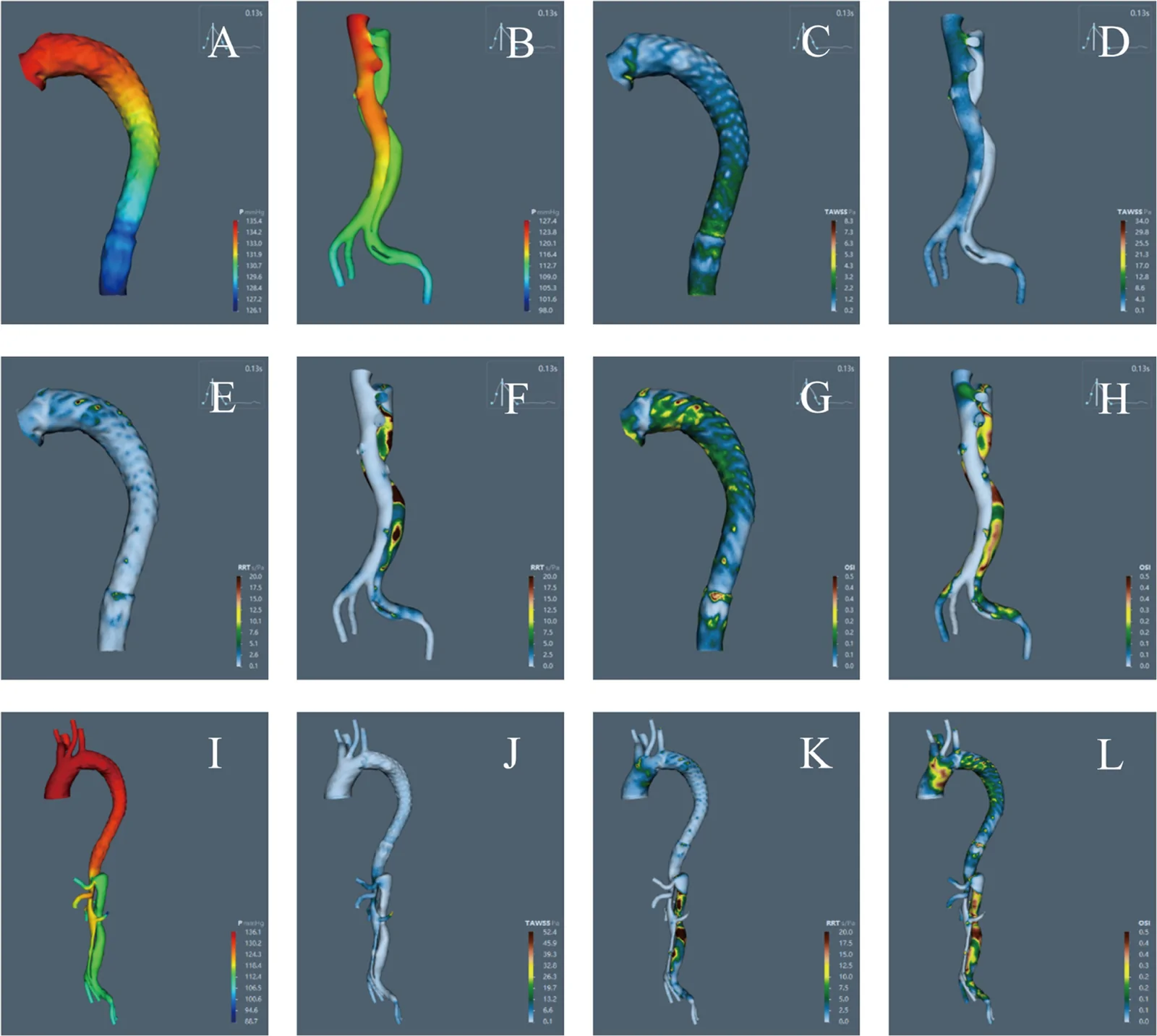
Based on S1 section, S2 section and the overall, calculate the average pressure (**A**, **B** and **I**), TAWSS (**C**, **D** and **J**), RRT (**E**, **F** and **K**) and OSI (**G**, **H** and **L**) for each section and the overall. TAWSS, Time-Averaged Wall Shear Stress; OSI, Oscillatory Shear Index; RRT, Relative Residence Time

Table 2; Fig. 5 present a quantitative analysis of hemodynamic parameters in the S1 and S2 segments of the aorta, as well as the overall aorta, before and after PMEGs, alongside blood flow in the visceral branch arteries. After PMEG treatment, aortic pressure showed a decreasing trend in the S1 segment, S2 segment, and the overall aorta. In the S1 segment, pressure decreased from 109.2 ± 20.3 mmHg preoperatively to 98.7 ± 17.4 mmHg postoperatively. In the S2 segment, pressure decreased from 97.6 ± 18.4 mmHg preoperatively to 90.7 ± 19.4 mmHg postoperatively, while overall aortic pressure decreased from 103.5 ± 19.4 mmHg preoperatively to 94.1 ± 17.8 mmHg postoperatively. However, none of these changes were statistically significant. After the operation, overall aortic TAWSS increased significantly, rising from 1.39 ± 0.50 Pa preoperatively to 1.78 ± 0.52 Pa postoperatively, with a statistically significant difference (*P* = 0.007). The findings from the segment-by-segment analysis revealed a significant increase in TAWSS in the S2 segment, from 1.33 ± 0.47 Pa preoperatively to 2.00 ± 0.89 Pa postoperatively (*P* = 0.005). In the S1 segment, TAWSS decreased from 1.08 ± 0.48 Pa preoperatively to 0.96 ± 0.32 Pa postoperatively, although this change was not statistically significant (*P* = 0.149).

**Table 2:** Comparison of the pressure, TAWSS, OSI and RRT at S1 segment, S2 segment and overall before and after PMEGs

	Pre-PMEGs	Post-PMEGs	*P*
Pressure (mmHg)			
S1 segment	109.2 ± 20.3	98.7 ± 17.4	0.077
S2 segment	97.6 ± 18.4	90.7 ± 19.4	0.238
Overall	103.5 ± 19.4	94.1 ± 17.8	0.106
TAWSS (Pa)			
S1 segment	1.08 ± 0.48	0.96 ± 0.32	0.149
S2 segment	1.33 ± 0.47	2.00 ± 0.89	0.005
Overall	1.39 ± 0.50	1.78 ± 0.52	0.007
OSI			
S1 segment	0.12 (0.09–0.14)	0.11 (0.08–0.15)	0.327
S2 segment	0.12 ± 0.04	0.09 ± 0.05	0.117
Overall	0.12 ± 0.03	0.10 ± 0.02	0.010
RRT (s/Pa)			
S1 segment	1.9 (1.4–3.3)	1.7 (1.2–2.7)	0.035
S2 segment	8.5 (3.5-2.5e8)	1.5 (1.1-2.0)	0.001
Overall	10.6 (3.2-9.1e8)	1.4 (1.2–2.2)	0.000
Blood flow (ml/s)			
Celiac trunk artery	36.1 ± 14.9	29.9 ± 21.6	0.309
Superior mesenteric artery	32.2 ± 11.2	40.2 ± 12.5	0.005
Right renal artery	21.7 ± 8.9	18.7 ± 7.5	0.251
Left renal artery	19.9 ± 10.4	17.7 ± 7.7	0.295
Volume (cm^3^)			
True lumen	34.5 (21.1–51.8)	36.1 (22.3–65.6)	0.845
False lumen	38.9 (19.4–68.7)	28.4 (14.3–58.1)	0.000

*PMEGs* physician-modified stent grafts, *TAWSS* Time‑Averaged Wall Shear Stress,* OSI* Oscillatory Shear Index, *RRT* Relative Residence Time

Following PMEG implantation, the overall OSI of the aorta decreased significantly from 0.12 ± 0.03 before the operation to 0.10 ± 0.02 after the operation (*P* = 0.010), indicating a significant reduction in the blood flow oscillation at the aortic wall. Segmental analysis showed a decrease in OSI in both regions, although without statistically significant differences. In the S1 segment, OSI decreased from 0.12 (0.09–0.14) preoperatively to 0.11 (0.08–0.15) postoperatively, while in the S2 segment, it decreased from 0.12 ± 0.04 preoperatively to 0.09 ± 0.05 postoperatively. Blood flow in the superior mesenteric artery increased significantly after PMEG treatment, from 32.2 ± 11.2 mL/s before the operation to 40.2 ± 12.5 mL/s after the operation (*P* = 0.005). In contrast, blood flow in the celiac trunk artery, right renal artery, and left renal artery showed decreasing trends, from 36.1 ± 14.9 mL/s preoperatively to 29.9 ± 21.6 mL/s postoperatively, 21.7 ± 8.9 mL/s preoperatively to 18.7 ± 7.5 mL/s postoperatively, and 19.9 ± 10.4 mL/s preoperatively to 17.7 ± 7.7 mL/s postoperatively, respectively; however, none of these changes were statistically significant.

### Aortic remodeling

In all 30 patients, an assessment of aortic remodeling was conducted using CTA before surgery and at 1-year follow-up. Figure 2 shows pre- and postoperative CTA images from a single patient, while changes in the TL and FL volumes are presented in Table 2. After surgery, the volume of the TL of the aorta showed an increasing trend, from 34.5 (21.1–51.8) cm³ to 36.1 (22.3–65.6) cm³, although this difference was not statistically significant. Regarding the FL volume, there was a significant decrease from 38.9 (19.4–68.7) cm³ before surgery to 28.4 (14.3–58.1) cm³ after surgery (*P* < 0.001).

## Discussion

This study investigated the management of patients with TBAD with residual distal dissection following initial TEVAR using staged PMEG interventions. The findings suggest that PMEGs effectively occlude distal entry tears while reconstructing TL morphology, thereby preserving perfusion to the visceral branches of the abdominal aorta. Furthermore, the systematic evaluation of hemodynamic changes and aortic remodeling outcomes provides a hemodynamic rationale for the clinical application of PMEGs to treat complex distal dissections.

CFD has been widely used to evaluate the hemodynamic characteristics of TBAD [16, 17], yet the impact of PMEGs on hemodynamics after closure of distal entry tears deserves further investigation. In Stanford type B aortic dissection, initial TEVAR effectively seals the proximal primary entry tear and reduces the risk of rupture in the acute phase, but it often fails to prevent persistent FL perfusion in patients with residual dissection extensively involving distal visceral branches. This persistent perfusion can contribute to long-term adverse events, such as FL expansion, the formation of dissecting aneurysms, and visceral ischemia [4, 18–20]. In this study, all patients underwent secondary PMEG intervention due to FL progression (83.3%) or post-dissection aneurysm formation (16.7%) during follow-up. The results demonstrated postoperative reductions in overall aortic pressure, as well as in the S1 and S2 segments, along with improved perfusion pressure in the visceral branch regions compared with preoperative levels. These findings indicate that PMEGs can effectively balance pressure distribution between the thoracic and abdominal aorta, reduce the high-pressure burden on the diseased vessel wall, and enhance perfusion to branch vessels, thereby establishing a hemodynamic basis for reducing the long-term risks of rupture and visceral ischemia.

This study found a significant postoperative reduction in OSI in the aorta, indicating decreased blood flow oscillation and a lower risk of endothelial injury. These findings corroborate the role of PMEGs in ameliorating hemodynamic disturbances and creating a more favorable mechanical environment for positive vascular remodeling [21–24].Postoperative TAWSS increased, most notably in the distal aortic segment (S2). Within the context of overall hemodynamic improvement, this change is consistent with flow normalization and remained within the broadly cited physiological TAWSS range of 1–7 Pa [25]. However, because the S2 segment coincides with the site of physician-modified stent alteration, the localized elevation should be interpreted cautiously, as altered flow dynamics at a structurally compromised stent segment may carry a long-term risk of device-related complications [21, 26–29].Therefore, long-term follow-up monitoring is warranted. Regarding RRT, previous studies have linked elevated values to increased thrombogenic potential [30–32]. Preoperatively, high RRT areas were predominantly localized within the FL, indicating blood stasis and a propensity for thrombosis. Postoperatively, RRT regions became more evenly distributed, but area of high RRT persisted within the stented segment, implying a potential residual risk of stent-related thrombosis. In conclusion, PMEGs have been shown to effectively optimize aortic hemodynamic conditions. These outcomes warrant close postoperative surveillance and the consideration of appropriate antiplatelet or anticoagulant therapy. Hemodynamic analysis may also provide critical insights for both surgical planning and postoperative pharmacological management.

In terms of aortic remodeling, the 1-year postoperative follow-up revealed a significant reduction in FL volume compared with the preoperative baseline, accompanied by an increasing trend in the TL volume. These findings confirm that PMEGs effectively obstruct distal FL perfusion, facilitate FL thrombosis and retraction, and thereby promote positive aortic remodeling. These outcomes are consistent with previous studies [3, 33]. Moreover, these structural changes are corroborated by corresponding hemodynamic improvements; the reduction in pressure load and restoration of wall shear stress collectively contribute to the inhibition of FL expansion and accelerate thrombus organization. This further reinforces the clinical value of PMEGs in managing residual dissections and preventing disease progression. In addition, postoperative blood flow in the superior mesenteric artery significantly increased, while perfusion to the remaining visceral branches remained stable, suggesting that PMEGs can effectively preserve visceral branch perfusion and do not significantly increase the risk of visceral ischemia, thus supporting their clinical safety and efficacy during reconstruction of the aortic morphology.

Whilst these findings highlight promising outcomes following PMEG treatment, this study has several limitations. First, as a single-center retrospective analysis with a small cohort of 30 patients, the statistical power to detect intergroup differences in certain parameters was limited, which may have introduced potential bias. However, the recruitment of a larger sample size remains challenging, as PMEGs are generally not routinely applied for the treatment of distal type B aortic dissection rupture. Second, the follow-up period was limited to one year, precluding an assessment of mid- to long-term outcomes (3–5 years and beyond), including durability of aortic remodeling and late complications. In addition, patient-specific hemodynamic data were not available; consequently, the inlet flow waveform was derived from the literature rather than from individual measurements. In addition, we did not perform a formal sensitivity analysis of inlet waveform or outlet Windkessel parameters. Future studies should incorporate patient-specific flow measurements and sensitivity analyses of boundary-condition assumptions to further validate the robustness of the hemodynamic findings. Finally, for computational efficiency, a rigid-wall assumption was used in the CFD simulations. Although this approach has been previously validated using 4D phase-contrast MRI [29, 34], further improvements in modeling accuracy are still necessary. In the future, this can be achieved by conducting fluid-structure interaction analyses to provide more detailed and accurate evaluation of aortic biomechanics and hemodynamic function.

## Conclusions

This study evaluated the efficacy of the PMEG technique in the treatment of distal dissection progression in TBAD, utilizing hemodynamic analysis to assess therapeutic outcomes in this complex aortic pathology. The results indicate that the PMEG technique significantly improves hemodynamic conditions in the thoracic and abdominal aorta, mitigates blood flow oscillation and stasis. Additionally, this approach effectively promotes FL thrombosis, decreases FL volume, and supports favorable aortic remodeling, while maintaining adequate perfusion to important visceral branches, such as the celiac artery, superior mesenteric artery, and bilateral renal arteries. However, the increased postoperative TAWSS warrants long-term follow-up observation.

## Data Availability

Due to the retrospective nature of this study and the potential risk of identifying individual patients, the raw data underlying this article cannot be shared publicly. The data were obtained from the Third People’s Hospital of Chengdu and are available from the corresponding author upon reasonable request, subject to approval by the hospital’s ethics committee review board and compliance with relevant data protection regulations.

## Abbreviations

3D: three-dimensional
CTA: computed tomography angiography
CFD: computational fluid dynamics
COPD: chronic obstructive pulmonary disease
CT: computed tomography
FL: false lumen
MRI: magnetic resonance imaging
OSI: oscillatory shear index
PMEGs: physician-modified stent grafts
RRT: relative residence time
S1: first operation segment
S2: second operation segment
TAWSS: time-averaged wall shear stress
TBAD: type B aortic dissection
TEVAR: thoracic endovascular aortic repair
TL: true lumen
WSS: wall shear stress
